# Targeting archetypes of viral-driven cancers with immunotherapy: a perspective on immunogenicity within the tumor microenvironment

**DOI:** 10.3389/fimmu.2025.1631258

**Published:** 2025-08-29

**Authors:** Keene Lee, Seohyun Kim, Junzhe Zhao, Shi Yong Neo

**Affiliations:** ^1^ Department of Otolaryngology—Head and Neck Surgery, Stanford Cancer Institute, Institute for Stem Cell Biology and Regenerative Medicine, Stanford University School of Medicine, Stanford, CA, United States; ^2^ Singapore Immunology Network, Agency for Science, Technology and Research, Singapore, Singapore; ^3^ A*STAR Skin Research Lab, Agency for Science Technology and Research, Singapore, Singapore; ^4^ Cancer and Stem Cell Biology, Duke-NUS Medical School, Singapore, Singapore

**Keywords:** viral-driven cancer, immunotherapy, immunogenicity, immune check inhibitor (ICI), tumor microenvironment (TME)

## Abstract

Viral etiologies of cancers have been widely studied for tumorigenesis and in recent years, widely recognized for their potential influence on immune regulation and response to immune checkpoint blockade (ICB). Here, we review the current understanding of how various oncogenic viruses are related to tumor immunogenicity and the tumor immune microenvironment. The present work also highlights the distinct features of these viral-driven cancers, that can be largely prognostic for better patient survival and response to ICB. On the other hand, there are also several commonalities in which these cancers acquire resistance against conventional immunotherapy. Finally, we discuss our perspectives to address the existing conundrums to gain clearer insights on how the interplay between anti-viral and anti-tumor immunity can be exploited to develop novel therapeutic interventions.

## 1 Introduction

Across different types of cancers, some of the key determinants of successful cancer immune checkpoint blockade (ICB) therapy often include tumor immunogenicity and the tumor microenvironment (TME) (1). Immunogenicity refers to the ability to induce adaptive immune responses influenced by the presence of tumor-derived antigens, and among these, neoantigens have gained increasing attention for its potential in enhancing anti-tumor immunity (2, 3). Neoantigens are novel peptides arising from somatic mutations such as single-nucleotide variants (SNVs), base insertions and deletions (INDELs) and gene fusions (4). Importantly, these unique peptides are highly immunogenic as they are not present in normal cells and thus are not subjected to central immune tolerance (5). A high tumor mutational burden (TMB) is generally associated with increased likelihood of generating immunogenic neoantigens, which is well-recognized as a strong predictor for response to ICB across cancer types (6–8). However, this relationship does not hold true across all cancer types. Cancers such as the viral-driven Merkel cell carcinoma (MCC) have low to moderate TMB yet reported to have high objective response rates (ORRs) to ICB (1, 9–11). In addition, the patient-specific nature of neoantigens results in a variable pre-existing T cell landscape which may also influence the eventual responses to ICBs (12). Characteristics of the T cell receptor (TCR) repertoire, such as clonal diversity, expansion and convergence may also serve as potential predictors of ICB treatment outcomes (13). It is noteworthy that TCRs generally have a much higher affinity for viral antigens than for tumor-related antigens (14). This critical finding could explain the inadequate efficacies of tumor antigen vaccines in priming T cells within the tumor-bearing host while at the same time providing a strong motivation for engineering TCR-based therapies for viral-associated antigens in cancers.

At the same time, understanding how the immune landscape is shaped within the tumor microenvironment (TME) plays a critical role in guiding immune intervention and developing innovative therapeutic strategies to target different tumor types. The immunological state of the TME can be broadly classified as “hot” or “cold”, which further modulates immunogenicity over the course of tumor progression. “Hot” tumors are highly inflamed and usually characterized by high infiltration of immune cells, particularly cytotoxic T cells, and increased expression of inflammatory markers such as IFN-γ and TNF-α (15). Typically, “hot” tumors are also associated with a higher TMB leading to elevated neoantigen presentation and better responses to ICBs (16). Moreover, certain cancers with “hot” TMEs can contain ectopic lymphoid aggregates commonly known as tertiary lymphoid structures (TLS) (17). These TLS are typically characterized by a central B-cell zone surrounded by a T-cell rich region, along with dendritic cells (DCs) and high endothelial venules (18). Depending on its maturation state, TLS can also contain activated B cells capable of differentiating into plasma cells that secrete high-affinity antibodies, which can enhance the anti-tumor immune response (19). In contrast, “cold” tumors are characterized by poor immune infiltration, lower TMB and PD-L1 expression. A “cold” TME is dominated by immunosuppressive cytokines such as IL-10 and TGF‐β, rendering them more unresponsive to existing immunotherapies (20). While this phenotype may vary across different cancer types, “cold” tumors generally represent an immune desert which do not respond well to ICBs. As such, various strategies are being explored to alter the immunological “temperature” of cold tumors and improve their response to immunotherapies (21).

**Figure 1:**
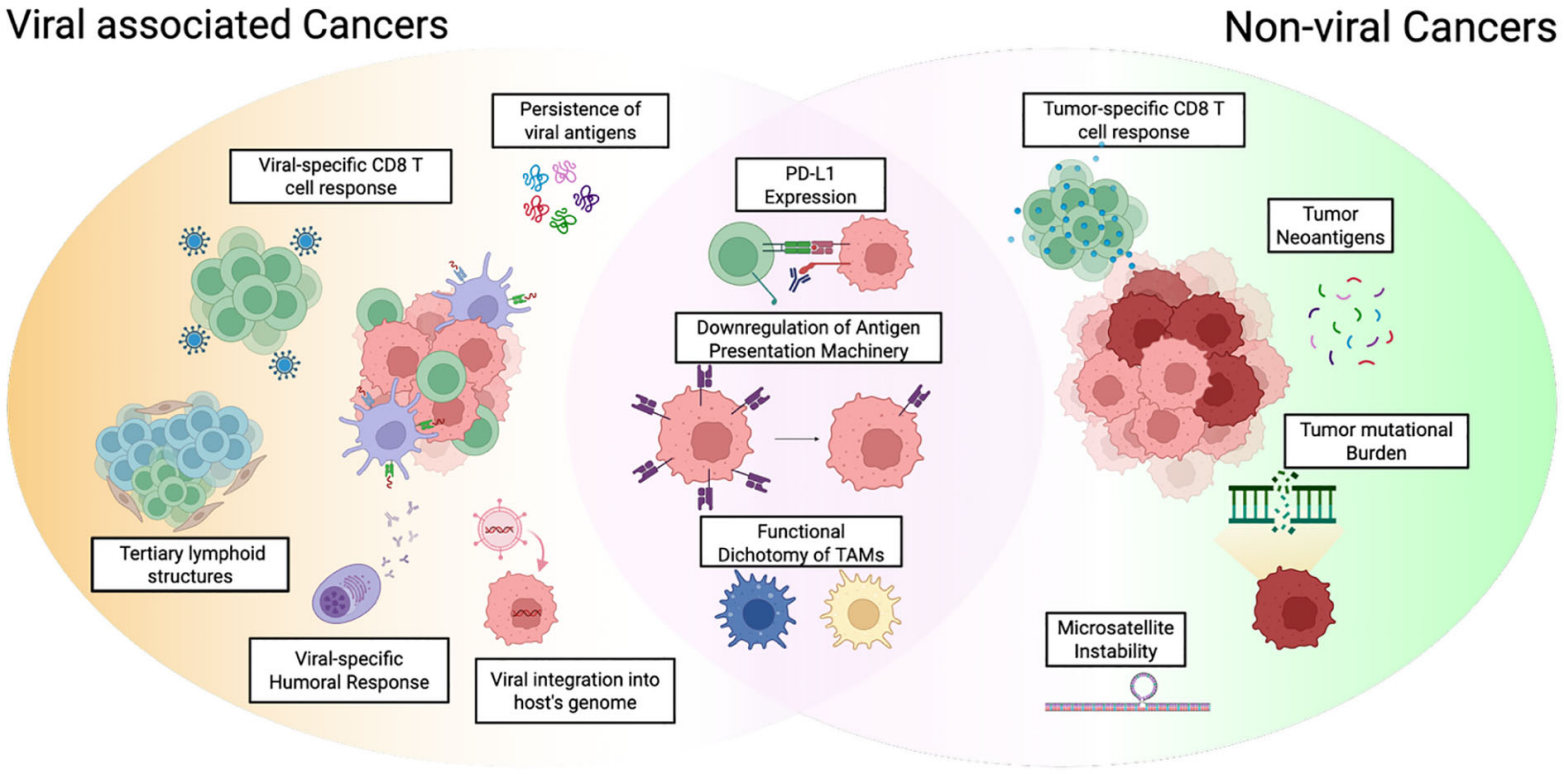
Summary of the key similarities and differences of the tumor immune microenvironment between viral and non-viral cancers. Factors generally more strongly associated with viral-associated cancers are depicted in yellow (Left), and those more strongly associated with non-viral cancers are depicted in green (Right). Similarities are shown in pink. In brief, viral-specific CD8 T cells are robust to mount a satisfactory anti-tumor immune response. Viral integration into host genome can influence immunogenicity and at the same time, potentially implicates the development of intrinsic tumor immune evasive mechanisms. While the TME can be favorably conditioned by the presence of TLS, there is a functional dichotomy of immune cells. In particular, tumor-associated macrophages (TAMs) and B cells can acquire either anti- or pro-tumoral immunoregulatory phenotypes to shape the immune landscape. Schematic illustration created with BioRender.com.

It is important to note that not all high TMB tumors are immunogenic or “immune-hot”. Consequently, such associations can only be applied to specific tumor types (22). Of interest, a recent meta-analysis reported cancers associated with HPV and HBV/HCV can be prognostic for better overall survival and even higher ORRs to ICBs (23). However, there is limited understanding on how viral factors modulate anti-tumor responses within the tumor immune landscape. As such, the present review discussed the underpinning research to identify how oncogenic viral factors play an integral role in influencing tumor immunogenicity and TME. Here, we focused on several cancer types that show great potential with ICB therapies and outlined common distinct traits across viral-driven cancers that impact anti-tumor immunity (Summarized in **Figure 1**). Ultimately, we deem that there is great potential to advance the immune oncology field towards harnessing anti-viral immunity across multiple types of viral-driven solid tumors.

## 2 Merkel cell carcinoma as a unique viral-driven neuroendocrine tumor

Merkel cell polyomavirus (MCPyV) has recently emerged as an oncogenic virus, accounting for at least 80% of the Merkel cell carcinoma (MCCs) cases worldwide. MCC is a rare and aggressive type of skin cancer with an exponentially increasing incidence rate between 2000 and 2013, highlighting the growing clinical importance of this disease (24). MCC is divided into 2 distinct molecular subtypes. Virus-negative MCC (MCPyV- MCC) is characterized by a higher mutational burden, with most of them resembling UV-induced mutations, while MCPyV+ MCC expresses primary oncogenic drivers small T (sT) and long T (LT) antigens (25). TMB was reported to be higher in MCPyV- MCC tumors as compared to MCPyV+ tumors (26). In fact, MCPyV-MCC tumors can harbor more tumor neoantigens than cutaneous melanoma or NSCLC (27). In a case study of a particular MCPyV-MCC patient, the presence of neoantigen-specific Th1 cells were detected after anti-PD-L1 therapy but intriguingly, no neoantigen-specific CD8 T cells were detected (28).

### 2.1 Immunogenicity of viral associated Merkel cell carcinoma does not correlate with tumor mutational burden

The high immunogenicity of MCPyV+ MCC tumors could perhaps be better explained by the presence of MCPyV antigens and viral-reactive T cells in ICB responders. The presence of KLL- (dominant epitope of the MCPyV common T-ag) specific intratumoral T cells favored better disease-specific survival and lower risk of metastatic disease (29). Frequencies of MCPyV-specific CD8 T cells also correlated with T-Ag antibody titers alongside increasing tumor burden (30, 31). Of interest, these viral specific CD8 T cells also highly expressed both PD-1 and TIM-3 which can be inhibited to improve T cell activation as demonstrated *in vitro* (31). Moreover, a traceable increase in T antigen-specific CD8 T cells was detected in the peripheral blood of ICB responders, which was associated with improved progression-free survival. The authors also further demonstrated that these viral reactive T cells can be expanded with robust killing capacity to target MCC tumor cells *in vitro* (32, 33). Another study critically uncovered that high baseline frequencies of circulating viral-specific CD8 T cells, but not intratumoral CD8 T cells, was associated with beneficial responses to ICBs, which may represent a potential application as a predictive biomarker for immunotherapy. Amongst MCPyV specific T cells, further CITE-seq (Cellular Indexing of Transcriptomes and Epitopes by Sequencing) analysis revealed intratumoral CD8 T cells to be terminally exhausted while circulating CD8 T cells highly expressed TCF7, CD62L and LEF1, resembling a more functional stem-like memory phenotype (34). Notably, tumor regression was achieved in a case reported where MCPyV-specific T cells were adoptively transferred (35). Collectively, these findings highlighted the potential of using other TCR-dependent therapies such as adoptive T cell therapy and tumor vaccines to complement the existing success of ICBs in treating MCCs.

Alongside the presence of these viral antigens, MCPyV+ MCC can promote immune evasion via multiple mechanisms. Like many other cancers, modulation in expression levels of MHC and other molecules of the antigen presentation machinery (APM) are widely implicated in the escape of immune surveillance in MCPyV+ MCC (36). Ritter et al. demonstrated that APM genes are epigenetically silenced by histone hypoacetylation, highlighting the potential use of HDAC inhibitors as therapeutic primers for response to ICBs (37). Likewise, the STING pathway (innate immune regulator stimulator of IFN genes) was also revealed to be largely silenced in MCC, implicating the downregulation of NF-κB signaling. The reactivation of STING could reconfigure the “cold” TME of MCC to enhance immune infiltration and surveillance (38, 39). Additionally, MCPyV sT was uncovered to interfere with type I IFN signaling by either direct transcriptional repression or histone modifications (40). Overall, it is evident that MCPyV can enhance immunogenicity and at the same time, drive immune tolerance within the TME of MCC.

### 2.2 Exploiting the favorable immune landscape of MCPyV+ MCCs for immunotherapy

Interestingly, MCC can be well-infiltrated with both effector and central memory T cells (41). In fact, MCPyV positivity in MCC is associated with greater infiltration of T cells and macrophages within the tumor microenvironment, which consequently contributes to favorable survival outcomes (42). Additionally, a gene signature derived from a comprehensive characterization of pro-inflammatory γδ T cells was found to be a potential predictor of improved survival and responses to ICBs (43). While most immunological studies on MCC were heavily dependent on phenotypic profiling of patient tissues, the engraftment of MCC tumor fragments into immuno-deficient NSG mice has been explored as a functional patient-derived xenograft (PDX) model. Comparing the phenotype of tumor-infiltrating lymphocytes (TILs) before engraftment to those 5 days post implantation into the mice, there is an increase in activated effector T cells and a reduction of FOXP3+ regulatory T cells (Tregs). The selective depletion of CD25+ T cells intriguingly enhances tumor growth *in vivo*, suggesting the presence of active T cell-mediated anti-tumor immunity that limited the initial growth of the engraft in the PDX model (41). However, there remains no evidence of Treg mediated impedance of anti-tumor immunity within MCC. Likewise, there could be potential involvement of humoral immunity in MCC. While antibody reactivity to MCPyV capsid protein VP1 is seemingly ubiquitous within the general population, the abundance of antibodies recognizing MCPyV tumor-associated oncoproteins (T antigens) was found to correlate with tumor burden. Importantly, the increase in titers of such antibodies precedes recurrence and metastatic progression, highlighting their potential as biomarkers for clinical utility (30). Future studies should be focused on further dissecting the understanding of B cells within MCC using deep immune-profiling to provide potential insights on how humoral immunity can be further exploited to improve conventional therapies.

### 2.3 Future prospects for immunotherapy in MCPyV+ MCC

Although ORRs reported with ICB treatment of MCPyV+ MCC is undeniably promising (44, 45), the direct interplay between MCPyV and the host’s immune system is not clearly understood. Moreover, treatment responses are greatly influenced by immune evasion mechanisms and the composition of the TME (46). Hence, further investigation into these factors is essential to uncover the novel biological insights and translatable therapeutic options, particularly for treatment-resistant patients. Interestingly, PD-1 expressed on MCC tumor cells was also demonstrated to be a growth promoter driving mitochondrial respiration and tumor cell proliferation, which can be reversed by conventional inhibition of the PD-1/PD-L1 axis (47). Considering the rare demographics of MCC, addressing further complex biological questions could be impacted by the limited motivation of scientists and oncologists. The fact that PD-L1 correlated with both MCPyV positivity and the infiltration of TILs (48) regardless highlights the value of leveraging MCC as a suitable model to further dissect new mechanisms underlying the cross talk between anti-viral and anti-tumor immunity in future studies.

At the same time, there are also some interests in developing tumor vaccines to target MCPyV+ MCC, though the current progress from such studies are largely preclinical. Early studies from more than a decade ago have already demonstrated the effectiveness of using DNA vaccines to generate LT-specific CD8 T cell responses in syngeneic tumor mice models (49, 50). Truncated LT can also be incorporated into monocyte-derived dendritic cells, which act as antigen presenting cells for the stimulation of autologous T cells (51). A more recent study further improvised a fusion design that integrates LT to LAMP1 (lysosomal-associated membrane protein 1), enhancing antigen presentation to potentiate antigen-specific CD4 T cell responses and humoral responses *in vivo* (52). Likewise, there are similar studies to evaluate the efficacy of DNA vaccines encoding sT, mounting antigen-specific CD8 T cell responses (53). However, it should be emphasized that the majority of these vaccine studies were heavily reliant on the overexpression of T-ag in cutaneous B16F10 melanoma cells prior to inoculation into immunocompetent mice. The use of alternative transgenic mouse models for MCC has been recently developed, which should be explored for future immunological studies (54). To delve deeper into the TME for new discoveries, one can also start with interrogating publicly available single cell sequencing of MCC tumors or perform immune gene deconvolution within bulk transcriptomics datasets (55, 56). With clear evidence of MCPyV-associated humoral immunity in MCC (30), another plausible hypothesis could be the potential involvement of B cells in the co-stimulation of T cells within the tumor-bearing host. However, to further dive deeper into understanding such immune regulation would require an immunocompetent mouse model – one that is currently lacking in the field.

## 3 Distinct viral-associated immune landscapes in hepatocellular carcinoma

Hepatocellular carcinoma (HCC) is the leading primary malignancy of the liver and the third most common cause of cancer-related mortality globally (57). 5-year survival is at a dismal 10-20% despite treatment. It typically arises in the background of chronic liver disease and cirrhosis. Risk factors of HCC include chronic viral (HBV/HCV) hepatitis, aflatoxin exposure, alcoholism, and metabolic syndrome, which features metabolic dysfunction-associated steatotic liver disease (MASLD) and metabolic dysfunction-associated steatohepatitis (MASH). Globally, HBV is responsible for more than 50% of HCC cases, especially in endemic regions such as Asia and sub-Saharan Africa (58). Notably, MASLD/MASH accounts for half of the new HCC cases in the US and, with the rollout of universal HBV vaccination, this trend is expected to take predominance in both industrialized and developing societies alike (59).

### 3.1 Etiology of HCC impacts immunogenicity and immune responses in HCC

HBV is a partially double-stranded DNA virus from the Hepadnaviridae family. It integrates into the host genome and expresses viral proteins such as HBsAg and HBx, contributing to oncogenesis via TERT or MLL4 (60) and to the modulation of immune surveillance (61). In contrast, HCV is a positive-sense RNA virus from the Flaviviridae family that does not integrate but causes HCC through sustained inflammation, fibrosis, and immune perturbation (62). Both viruses can shape distinct immune microenvironments, affecting tumor development and treatment response. As such, the immunogenicity of HCC can vary with its underlying cause. In HBV-related HCC, the presence of viral antigens, including HBsAg, HBcAg, and HBx, theoretically provides targets for immune recognition of the tumor; but it is often HBx that remains as the only HBV protein detectable in tumor cells (63). Notably, HBx promotes tumor invasion and metastasis in a mechanism unrelated to diminished immunogenicity (63). Virus-specific CD8+ T cells are detectable in peripheral blood and TILs in HBV-HCC patients (64–66). However, these T cells are often functionally exhausted due to persistent antigen stimulation, high expression of inhibitory receptors (e.g., PD-1, LAG-3), and metabolic dysregulation (67). Similarly, another inhibitory receptor, TIGIT was also upregulated by TILs and the co-inhibition with PD-1 could restore immune activation ex vivo (68). Together, these evidence offers an explanation towards the limited efficacy of HBV-specific T cell therapy in HCC control (69–72). While HCV-related HCCs also present viral antigens capable of stimulating T cell responses (73), there is limited literature regarding the presence of HCV-specific T cells in the TILs of HCV-HCC patients. MASLD-HCC, however, is more reliant on neoantigen-driven immunity. Notably, TMB is often higher in non-viral HCCs (especially MASLD-HCC), potentially increasing the number of neoantigens presented by tumor cells (74). Despite a higher TMB, MASLD-HCCs do not uniformly exhibit robust immune activation, suggesting that antigen presentation or immune cell priming may be impaired.

### 3.2 Influence of the tumor microenvironment may play a larger role than immunogenicity during treatment of HCC

The composition and functional state of the TME in HCC are shaped by its etiology and play a crucial role in determining prognosis and response to immunotherapy. A detailed analysis of lymphoid and myeloid populations reveals both conserved and aetiology-specific features. In HBV-related HCC, the TME is enriched with immunosuppressive populations, especially Tregs, tumor associated macrophages (TAMs), and myeloid-derived suppressor cells (MDSCs) (67, 75–77). Tregs can contribute to local immunosuppression and correlated with worse prognosis in HCC patients (78). CD8+ T cells are often excluded from the tumor core, a phenomenon correlated with TGF-β signaling and the presence of activated hepatic stellate cells (79). TAMs, on the other hand, are highly heterogeneous in origin or phenotype in HBV-HCC but typically possess immunomodulatory characteristics (80). In addition, IL-10-expressing B cells, present in HBV-HCC, suppresses CD4+ T cell activity (81). Lesser is understood about the tumor microenvironment of HCV-HCC tumors. A prominent feature uncovered is that chronic viral antigen stimulation drives CD8+ T cell exhaustion with elevated PD-1 and TIM-3 expressions (67, 82). These T cells often exhibit a downregulation of T cell activation signatures compared to those in HBV-HCC, which correlates with a reduced immune infiltration in HCV-HCC tumors (83). Of note, in chronic HCV-infected patients, NK cell expression of TIM-3 and CD38 may be an early sign of impending HCC (84). While there are studies that did not find differences in the proportion and phenotypes of TILs in HCV-HCC compared to other etiologies (85, 86), further investigations would be warranted, particularly in the understanding of immunoregulatory cell types within the TME.

Unlike viral-driven HCCs, the immune landscape of MASLD-HCC is markedly distinct. Early-stage MASLD is accompanied by increased macrophage and lymphocyte infiltration, where these macrophages are predominantly proinflammatory, engaging in TNFα and IL-6-based acute inflammatory responses (87). Importantly, a recent seminal paper showed that the activated PD-1+ CD8+ T cells in MASH do direct damage and resulted in impaired immune surveillance and HCC development (88). Prophylactic anti-PD1 ICI treatment in MASH mice resulted in increased, rather than decreased, HCC tumorigenesis (88). Back-to-back published, it was also shown that these activated T cells exhibit resident, effector, and exhausted characteristics, and perform killing functions independent of antigen presentation, resulting in liver damage commonly seen in MASH (89). In addition, there may be dominance of TREM2+, MARCO+, and CD206+ macrophages within tumor and peritumoral regions in MASLD-HCC (90). These macrophages have high lipid, impaired phagocytosis, and produce cytokines such as IL-6, IL-10 and TGF-β (91, 92). Moreover, CD8 T cells are often sparse and localized to the tumor margin, co-expressing PD-1 and CD39 with an exhausted phenotype (88, 93). Interestingly, cancer-associated fibroblasts (CAFs) are also enriched in inflammatory gene signatures and produce IL-34, which may promote Treg infiltration and suppress CD8+ T cell activity, especially in non-viral HCC (94, 95). These findings have highlighted the importance of inflammation in MASLD-HCC transition, and the distinct temporal roles of different immune cell populations in the liver during disease development and progression.

### 3.3 Understanding viral/non-viral immune evasion mechanisms for future therapeutic directions

Immune escape is central to HCC progression and varies according to etiology. In HBV-related HCC, viral proteins like HBx can downregulate MHC class I molecules and IFN-γ expression, impairing CD8+ T cell recognition and inducing their apoptosis (96, 97). Silencing HBx with a 5’-triphosphate siRNA can reduce the differentiation of Tregs and MDSCs (98). In HCV-related HCC, immune evasion arises from T cell exhaustion and altered antigen-presenting cell (APC) function (99). DCs in HCV-HCC also exhibit impaired IL-12 production, diminishing effective T cell priming (100). As such, engineering CAR-T cells and CAR-NK cells against known HCC targets like GPC3 might represent promising strategies to overcome these potential immune evasion mechanisms within the TME (101–105). In MASLD-HCC, immune suppression is contrastingly driven by metabolic dysfunction. Lipid accumulation in hepatocytes and immune cells impairs immunogenicity (106). TAMs, (including peritumoral macrophages and monocytes), upregulate PD-L1 (107, 108). IL-6 secreted by TAMs contributes to STAT3 activation in tumor cells, reinforcing immune resistance (109). Still, anti-PD-1/PD-L1 ICB therapies have transformed the treatment landscape for advanced HCC (110, 111). The profound clinical question is, therefore, what predicts the response to ICBs. While a recent study shows that poor Atezo/Bev response is associated with high glypican-3 (GPC3) or alpha-fetoprotein (AFP) expression, no correlation was seen between the expression of PD-L1 and Atezo/Bev response – contrary to the data in non-small cell lung cancer or melanoma (112). Notably, etiology may impact therapeutic outcomes. A recent meta-analysis of 3,739 patients shows that non-viral HCC does not seem to benefit, or benefit less, from ICBs compared to HBV-HCC, yet the heterogeneity of the trials is high (113, 114). This underscores the need to dissect the immunological nuances across HCC etiologies.

Indeed, targeting the immunosuppressive TME also represents a promising avenue for improving outcomes in HCC. Combination therapies that integrate ICB with multi-kinase inhibitors (e.g., cabozantinib) have shown promise in clinical trials, especially for the HBV-HCC subgroup (115, 116). Furthermore, several trials also investigated the potential of an oncolytic viral vaccine, Pexa-Vec as means to completement conventional HCC therapies (117–119). These regimens may leverage immunogenic cell death and inflammation to enhance ICB response. Myeloid-targeted therapies such as anti-TREM2, anti-MARCO, and CSF1R inhibitors are also under preclinical investigations, which may synergize with conventional ICBs (120–122). However, inhibiting TREM2 or depleting TREM2+ macrophages may have repercussions for their protective functions particularly at the earlier stages of liver damage (123–125). Similar dual protective-immunosuppressive functions also hold in MARCO (126–129). Therefore, different studies are disconnected in terms of the temporal progression of myeloid functions, where the proinflammatory and anti-inflammatory roles of myeloid cells may both promote HCC tumorigenesis and progression, depending on the rather heterogeneous temporal sequence of events. Importantly, new platforms including organoids can incorporate autologous immune cells and stromal components to offer powerful translational tools for biomarker discovery and drug screening in HCC (130). Their ability to retain patient-specific TME features, including lipid dysregulation and immune cell crosstalk, makes them ideal for preclinical testing of aetiology-specific immunotherapies (131). Future work should focus on refining preclinical models, validating findings in clinical cohorts, and integrating spatial and multi-omics approaches to fully map the immunologic heterogeneity of HCC (132). Stratifying patients by etiology and immune profile may enable more effective, personalized immunotherapeutic interventions.

## 4 Role of human papillomaviruses in carcinogenesis

Human papillomaviruses (HPVs) are a subclass of papillomaviruses, which are non-enveloped, icosahedral, double-stranded DNA viruses (133, 134). Although over 200 strains of HPV have now been identified, they can be generally stratified into high and low-risk HPVs – representing two subgroups of HPVs that are either overrepresented or rarely present in HPV-positive (HPV+) cancers (135). Of note are HPV16 and HPV18, the two most common high-risk strains of HPV in HPV+ cancers (136, 137). While most HPV infections are eventually cleared, chronic infections, particularly with high-risk strains, can result in the development of a range of anogenital and oropharyngeal cancers (OPCs). HPV is by far the leading cause of most anogenital cancers, accounting for 40-95% of vulvar, vaginal, penile and anal cancers and virtually all cervical cancers (137, 138). Furthermore, it accounts for around 70% of all OPCs and around 20% of other head and neck squamous cell carcinomas (HNSCCs) (139). HPV viral oncogenes E6 and E7 have been shown to be necessary and sufficient for cellular immortalization and transformation (140–142). Briefly, E6 acts to degrade p53 by associating with the E6-associating protein (E6-AP) – a canonical E3 ubiquitin ligase, coordinating with E7, which blocks binding of key cell cycle checkpoint proteins including pRb, p21 and p27, together causing deregulated cell cycling and genomic instability. This instability has been thought to be the main contributor to the slow kinetics of carcinogenesis, through gradual accumulation of genomic aberrations coupled with uncontrolled cell cycling.

### 4.1 HPV-associated immunogenicity may vary with host genome integration

Our discussion here will focus on HNSCCs, in which responses to immunotherapy have met much more variability. This is in contrast to cervical cancers – which are consistently reported to have high ORRs to ICBs (143–146). An important difference to note is that while virtually all cervical cancers are viral associated, only a subset of HNSCCs are HPV positive. HPV+ HNSCCs are generally associated with better prognoses compared to HPV- HNSCC, even after controlling for confounding factors such as tumor stage, smoking status and alcohol usage (147–150). Furthermore, they demonstrate enhanced radio and chemotherapy responsiveness compared to HPV- HNSCC (150). However, there are mixed results when comparing the efficacy of immune checkpoint blockade (ICB) between HPV+ and HPV- HNSCC cases, with some studies demonstrating enhanced response amongst HPV+ cases (149, 151, 152), and some demonstrating no difference (153). This discrepancy might be attributed to how the levels of PD-1/PD-L1 expression cannot be trivially disentangled from the level of immune infiltrate. Notably, there are also contradicting reports of PD-L1 and PD-1 levels and viral status, with some reporting increased levels in HPV+ HNSCC (154–157) and others reporting no correlation (151, 158–160). This is probably indicative of heterogenous cohorts and implies that PD-L1 levels are also influenced by other tumor intrinsic/extrinsic factors not related to viral status. Nevertheless, the expression of PD-L1/PD-1 stratifies responders to ICBs in HNSCC, which can be, in part, causally linked to viral status and immune infiltration (153, 155, 161).

It is important to note that in HPV+ cancers, the HPV genome can exist as an episome, integrated into the host genome or a mixture of both, although in most HPV+ cervical cancers (~80%), they are stably integrated into the host genome (162). This aberrant integration event often occurs within the coding region of the E2 gene, which codes for an important transcription factor that is essential in the careful regulation of the expression of the oncogenic proteins E6 and E7 (163, 164). Sustained E6 and E7 expression are essentially required for the establishment and persistence of HPV+ cancers (165, 166) and consequently, the genomic landscapes of HPV positive and negative cancers are vastly different (167–171). There are contrasting studies on the impact of viral status on mutational loads, with some demonstrating no difference (169), and some showing greater mutational burden in HPV- HNSCCs (172, 173). Despite this, the mutational landscape of HPV+ and HPV- HNSCCs vary widely due to the origins of these genomic aberrations.

Due to the presence of viral antigens and increased immune cell infiltration into the tumors, HPV+ HNSCCs are generally thought to be more immunogenic (174). Interestingly, the viral genome is often maintained in episomes or a mixture of integrated and episomal genomes in HPV+ HNSCCs (175, 176). A recent study looking at integration events in HPV+ HNSCCs revealed that integration-negative tumors correlated with an increased immune signature, specifically T, B and NK cells compared with integration-positive HPV+ HNSCCs (177). Furthermore, emerging evidence has demonstrated constant expression of many early viral genes when the genome is maintained episomally, drawing the link between integration-negative HPV+ HNSCC and increased viral antigenic presence to influence viral-specific immune responses (178, 179). Integration events also play a role in modulating the genomic landscape, gene expression profiles and even epigenetic signatures (180) within HPV+ HNSCCs, driving differential responses to various therapies and correlating with prognosis (181). Such events presumably also contribute to divergent TMEs between integration positive and negative HPV+ cancers (182). It is noteworthy that HPV+ HNSCCs rarely exhibit oncogenic/tumor suppressor driver mutations (183, 184), and instead are completely dependent on the E6/E7 viral oncogenes (165, 166). In the context of non-viral OPCs, tobacco and alcohol overuse are strong contributors of overall TMB and immunogenicity (168, 173, 185). Notably, immunogenicity and immune cell infiltration of HPV- OPCs vary widely, depending on neoantigen load and driver mutations accumulated (161, 186). Nevertheless, a higher TMB in HNSCC, independent of viral status, is linked to superior immunotherapy responses (161, 171, 187).

### 4.2 HPV-associated HNSCCs are largely influenced by their microenvironment

Similar to HCCs, the role of the tumor microenvironment of HNSCCs appears to be a stronger deterministic factor for ICB responses rather than immunogenicity. A high degree of intratumoral immune cell infiltrate may be a key factor in HPV+ individuals’ improved response to conventional treatment and favorable clinical outcome. In HNSCCs, the immune landscape of HPV+ tumors had considerably more infiltrating IFNγ+ CD8+ T lymphocytes, DCs and more proinflammatory cytokines within the milieu (157). Multiplex immunofluorescence coupled with immune-related gene expression profiling revealed that compared to HPV- OPCs, HPV+ lesions were more heavily infiltrated by CD8+ T cells, with an increase in various subsets of T cells including cytotoxic and exhausted cells. Spatially, these T cells appeared in much closer proximity to tumor cells, CD163+ macrophages and FOXP3+ Tregs (154). This overall suggests a stronger activation of immune pathways and an inflamed TME. Additionally, Eberhardt et al. identified CD8 T cell clones specific to a range of E proteins, and further characterized a subset of HPV-specific PD-1+ stem-like population capable of proliferating upon exposure to antigen *in vitro* (188). This study presents evidence of the ability of these T cell clones to maintain cytotoxic responses under persistent antigenic exposure and ultimately, alludes to the amenability of HPV+ HNSCC to respond to PD-1 checkpoint blockade.

While the differentiation trajectories of CD8 T cells are relatively similar between HPV+ and HPV- HNSCCs, there could be viral-driven divergence in the polarization of CD4 T cells and B cell subsets (189). Leveraging TCGA datasets, it was reported that HPV positivity correlated with increased levels of CD4 T follicular helper (Tfh) and Tregs (190). The CD4 T cell compartment in HPV+ samples also showed skewing towards an inflammatory Th1 response. Furthermore, these T cells presented with a higher expression of a range of exhaustion-related molecules including LAG3, PD1, TIGIT and TIM3, which counterintuitively correlated with improved survival, presumably suggesting an active T cell response. Importantly, this correlation was not seen in HPV- samples, indicating a viral-specific T cell response. This ‘T-cell-inflamed’ phenotype points towards the potential of immune checkpoint inhibitor blockade as a HPV+HNSCC-specific treatment. While inflammation could drive immune tolerance within the TME, it still remains unclear if Tregs are significantly enriched in HPV+ HNSCC compared to HPV- HNSCC. It appears that there is heterogeneity in localization of these Tregs, which some studies observing an increased Treg infiltrate in the stromal compartment (191), while others demonstrated enriched Treg signatures within the intraepithelial compartment (192). Studies have also reported correlation of a higher level of Treg infiltration with better prognosis in certain HNSCC subsets (193, 194). While Treg-dependent immune suppression is associated with poor prognosis in some cancers, the paradoxical opposite observed in HPV+ HNSCCs is thought to be reflective of an overall pro-inflammatory immune microenvironment, promoting general CD4/8 T cell infiltration (190). This immune ‘hot’ environment is speculated to be a virus-dependent phenomenon.

Shifting focus to viral-driven humoral immunity differences, while CD20+ B cells were enriched in HPV+ HNSCC, studies did not find correlation between B cells and patient survival (192, 195). However, the TME of HPV+ HNSCC is characterized by active HPV-specific intertumoral B cell responses and antibody production (196). While antibodies against viral proteins such as E2, E6 and E7 were detected, E2-specific responses appeared most dominant, based off IgG titers. The authors also demonstrated the preferential localization of these B cells (and antibody-producing cells) to the tumor stroma, where they form germinal center-like clusters indicative of an activated B cell phenotype. While the link between HPV-specific antibodies and enhanced anti-tumor immunity is unclear, studies have evidenced a correlation between anti-HPV antibodies and survival benefit (197, 198). Another study revealed that germinal center B cells were enriched in HPV+ HNSCC, while HPV- HNSCC had fewer total B cells and presented in a non-germinal center state (189). Further TCGA analysis also demonstrated enriched signatures of plasma and memory B cells in HPV+ HNSCC, which correlated with higher CXCL13 production from CD4+ T cells (199). This correlated with better prognosis, indicating viral-specific mechanisms driving preferential induction of TLS that presumably contributes to enhanced antitumor responses as a peripheral consequence of persistent HPV infections. Using a murine model of HPV+ HNSCC, Kim et al. demonstrated an expansion of memory B cells, plasma cells and antigen-specific B cells upon radiotherapy or PD-1 blockade. Furthermore, IgM and IgG serum levels were elevated post PD-1 treatment in a cohort of HPV+ HNSCC patients that showed positive clinical response, strongly suggesting that a similar mechanism of B cell expansion correlated with better response to PD-1 blockade (200). Collectively, the notion that B cells may play a key role in the immune-mediated eradication of HPV-driven HNSCCs is promising. It is therefore imperative to gain a better mechanistic understanding of this link, perhaps through established murine mouse models of HPV+ HNSCC, informing of potential vaccination strategies to enhance B cell responses against HPV.

Focusing on the innate lymphocyte compartment, particularly NK cells due to their heavy involvement in early anti-tumor and metastatic responses (201), a pan-cancer analysis uncovered HNSCCs to have one of the highest mean CD56^dim^ NK cell infiltration particularly in HPV+ HNSCCs compared to HPV- HNSCC (202, 203). This phenomenon could perhaps explain why lower MHC class I expression is correlated with favorable prognosis in HPV+ HNSCC but a poor prognosis in HPV- HNSCC (204, 205). NK cell activity is also modulated by the balance between activating and inhibitory ligands present within the TME (206). Of interest, HPV+ HNSCC trended towards a higher HLA-G expression than HPV- samples (192). HLA-G is a known inhibitory MHC molecule is recognized by KIRs and LILRB1/2 expressed on NK cells, which represents a possible NK inhibitory axis that is differentially modulated based on viral status of HNSCC (207). Interestingly, an intraepithelial ILC1-like NK state was also described in HNSCC independent of HPV status. These CD49a+CD103+ cells represented a tissue-resident (trNK) phenotype that co-expressed key cytotoxic signatures indicative of its ability to kill tumor cells (208). It would be of interest to dissect the impact of viral presence on the tissue-residency status of NK cells, since these trNKs represent a potentially immunomodulatory subset of NKs (209) that maintain the activation status of various adaptive immune cells, including CD4+ and CD8+ T cells (210).

While HNSCCs possess favorable features of adaptive immunity, the regulatory role of myeloid cells may play a critical influence as well. Increased infiltration of CD68+ macrophages is associated with poorer prognoses in HNSCC (211). Notably, there is an increased density of CD68+ macrophages in transcriptionally active HPV+ HNSCC compared to HPV- HNSCC patients. M1-like macrophage inflammatory signatures were also enriched in HPV+ HNSCC alluding to unique viral-driven mechanisms of modulating monocyte infiltration and macrophage polarization (190, 212). Similarly, the functionality of DCs within the TME can be influenced by viral-driven factors. Despite showing no correlation between the abundance of infiltrating plasmacytoid dendritic cells (pDCs) and viral status in HNSCC, pDCs have a reduced capacity to produce IFNα upon toll-like receptor activation in HPV-negative samples but remain uncompromised in HPV+ tumors. This effect was dependent on differential levels of TNFα and IL-10 between viral and non-viral cases (213). While no direct link between viral status and TNFα levels has been discovered yet, we speculate that this difference in cytokine milieu is attributed to the different forms of immune evasion mechanisms that the HPV virus takes during chronic infections and carcinogenesis of HNSCC compared to non-viral-induced mechanisms.

### 4.3 Addressing immune evasion and uncertainties in HPV-associated HNSCCs

There is clear evidence of viral-specific regulation within the TME of HNSCC. HPV-specific mechanisms of immune evasion in HNSCCs have been widely studied and previously discussed (214–216). In general, HPV early proteins (particularly E5, E6, E7) are central in the downregulation of host immune responses against the virus. Some common nodes of immunomodulation include modulation of the NF-κB pathway (217–220), inhibition of inflammatory cytokine production (221, 222), interferon and pattern recognition receptor signaling (217, 223, 224) and the disruption of antigen presentation processes (225–230). These mechanisms aim to downregulate the recognition and activation of innate and adaptive immunity against the virus. It is important to note that these evasion mechanisms are not exclusive to viruses capable of inducing carcinogenesis. These processes are, however, seen as factors that exacerbate carcinogenesis by inducing chronic inflammation due to persistence of HPV infections, since immunosurveillance is widely disrupted and viral clearance is impaired (231). Further mechanistic studies are needed to elucidate the relative contributions of viral evasion mechanisms to the suppression of anti-tumor immunity in these HPV-driven cancers.

Nevertheless, it appears that targeting these viral mechanisms could, in theory, be complementary to immunotherapeutic options (such as immune checkpoint blockade) against HPV+ cancers, since eliminating the virus would target the main vulnerability of these malignancies. Antigen-primed DCs were explored to mount HPV-specific responses to complement CAR-T cell therapy (232). Even in the absence of known antigens, such DC vaccines could also be prepared by the fusion of tumor and dendritic cells ex vivo (233). Importantly, robust pre-clinical and clinical responses to TCR-T cell therapy targeting HPV-E7 in HNSCC and cervical cancers were observed (234, 235). Still, prophylactic vaccinations against HPV strains is the current best option to prevent cervical cancers (236), which are almost always caused by a persistent HPV infection. While efforts are underway to determine the efficacy of therapeutic HPV vaccines against HPV+ HNSCC (237–241), it is imperative to better understand the consequences of chronic HPV infection on immunomodulation within the TME to determine if targeting the virus is a viable option as a therapeutic.

## 5 Epstein-Barr virus amongst the earliest known viruses for oncogenesis

Epstein-Barr virus (EBV), the first human tumorigenic virus discovered in 1964 in Burkitt’s Lymphoma cells, has since been implicated in various malignancies including epithelial cancers like gastric cancers (GCs) and nasopharyngeal carcinomas (NPCs) (242). Unlike the positive findings reported on HPV and HBV/HCV-associated cancers, the presence of EBV may not necessarily contribute to better OS or ORRs across EBV-driven cancers (23, 243). Our current understanding is that the involvement of EBV in the modulation of the tumor immune landscape is much more complex in contrast to other oncogenic viruses, which could explain the highly variable therapeutic responses to ICBs as reported in EBV-associated cancers.

Of which, EBVaGCs (EBV-associated gastric cancers) seem to yield better clinical outcomes despite being a small minority of EBV-driven cancers. EBVaGCs is a molecularly and clinically distinct subtype of gastric cancer accounting for about 10% of gastric cancers world-wide (244). It is largely driven by extensive viral epigenetic modifications, mainly DNA hypermethylation, unlike other gastric cancers, which are driven by mutational burden or genomic instability such as high microsatellite (MSI) or chromosomal instability (CI) (245). EBV promotes oncogenesis through BART miRNAs and BARF1 (BamHI-A rightward frame 1a), inducing methylation and altering gene expression. EBVaGC is not only characterized by high DNA hypermethylation but also frequent PIK3CA mutations and overexpression of JAK2, PD-L1 and PD-L2 (246). Paradoxically, despite its immune-rich TME, it exhibits reduced sensitivity to conventional chemotherapy (docetaxel and 5-fluorouracil) (247), highlighting the necessity for further investigation on its immunogenic profile and alternative therapeutic strategies such as immunotherapeutics for better patient outcomes.

### 5.1 EBV drives distinct immune profiles in gastric and nasopharyngeal carcinomas

Like other viral-driven cancers, the immunogenicity of EBVaGCs is also neither driven by mutational burden nor genomic instability. EBVaGCs were found to be mutually exclusive from MSI-H GCs with high amplification of PD-L1 expression (248–251). Conflictingly, PD-L1 expression can be either associated with both poor or better patient survival (250, 252, 253). While MSI-H tumors typically respond well to conventional ICBs, a case study reported a late stage metastatic EBV-GC patient that also showed beneficial response to PD-L1 blockade (avelumab). Of note, the patient’s tumor did not show high mutational burden or any mismatch repair defect. The authors then interrogated TCGA cohort showing that EBV-GC are microsatellite stable with low mutational burden but are well infiltrated by immune cells (254). Another study reported a patient with EBVaGC that displayed durable complete response to ICB, overcoming resistance to trastuzumab plus chemotherapy (255). While MSI-H tumors are associated with B2M (beta-2 microglobulin) mutation, which is a form of acquired resistance to immunotherapy, durable responses to ICBs can still be observed in MSI-H tumors within EBV-negative GCs (256). Furthermore, though not significant, a considerably high numbers of MSI-high GCs were either negative for HLA-A/B/C (22/37 cases) or B2M (21/37 cases) (257). Conversely, EBVaGCs highly express both MHCI and MHCII molecules, which is likely a potential consequence of being highly infiltrated by activated immune cells into the TME (257–259). In fact, the expression of HLA-DR was shown to be prognostic for better five-year overall survival (260). Importantly, EBVaGC represents a distinct clinicopathological entity with low incidence of lymph node invasion (249, 261). Following up by the same authors, EBVaGC tumors were reported to be better infiltrated with CD8 T cells and mature DCs (262). Both higher infiltrates of CD8+ and FOXP3+ cells were also found to be prognostic for better five-year overall survival (253). Likewise, the TME of EBVaGCs can contain high density of DCs, and interestingly, the maturation of these DCs can also be suppressed by exosomes derived from EBVaGC tumor cells (263).

Unlike gastric cancers, a great majority of NPCs are EBV+, which are also much more extensively studied given the considerably higher occurrences particularly across Asian countries (139, 264). Notably, United States was the second most common study sites for immunotherapy trials of NPCs despite much lower incidence rates across the world (243). Various EBV encoded nuclear antigens (EBNAs) and latent membrane proteins (LMPs) can be expressed in NPCs (265). Of which, EBNA1 can be overexpressed in NPCs, associated with metastasis (266) and immunosuppression within the TME (267). Mechanistically, EBNA1 contributes to TGFβ-mediated Treg formation and the production of Treg chemoattractants, CCL20 and CXCL12 (267, 268). Even though EBNA1 can be considered an EBV antigen, it is poorly immunogenic in cancer (269). Several other EBV-derived molecules were also found to play an integral role in tumor immune escape in NPCs. Like EBVaGCs, reports revealed mutations and downregulation of MHCI and MHCII molecules in EBVaNPCs (270, 271). Another EBV-encoded protein, BNLF2a, also inhibits TAP (transporter associated with antigen processing) to reduce antigen presentation and evade EBV-specific CD8 T cells (272). At the epigenetic level, LMP2A mediates hypermethylation of the HLA-ABC promoter. It was further demonstrated that the use of 5’-azacytidine as a demethylation agent was able to restore the expression of HLA-ABC in epithelial-origin tumor cell lines *in vitro* (273). In addition, EBV-encoded microRNAs (miRNAs) have profound immune suppressive effects against viral-specific T cells through means of downregulating of TAP1, TAP2 and HLA-ABC (274). Likewise, these EBV miRNAs also suppress the differentiation of naïve CD4 T cells into Th1 cells and the subsequent release of pro-inflammatory cytokines (275). Other non-coding RNAs such as circular RNAs (circRNAs) could also be involved in the immune modulation of the TME. EBV-encoded circBART2.2 was demonstrated to upregulate PD-L1 in NPC by promoting RIG-I signaling and the activation of IRF3 and NF-kB, causing T cell suppression (276).

### 5.2 Roles of tertiary lymphoid structures and B cells within the TME of NPCs

NPCs can be a promising target for ICBs considering the likely formation of TLS within the TME. Distinct TLS formation has been profiled in EBVaNPC, identifying a unique population of CXCL13-producing CD4+ T cells which can contribute to the recruitment of B cells and the maturation of TLS (277). Importantly, only B and plasma cells correlated with tumor mutational load in NPCs (278). Furthermore, in a recent study by Helmink et al., the enriched presence of B cells within TLS correlated with better outcomes in patients treated with ICB (279). While TLS can be common within NPC tumors, EBV-encoded LMP1 can suppress the maturation of antibody secreting cells and germinal center B cells. At the same time, LMP1-expressing B cells can act as regulatory B cells with high expression of IDO-1 (indoleamine 2,3-dioxygenase 1) (280). NPC-derived LMP1 was found to be non-immunogenic, in contrast to B cell-derived LMP1 which is capable of eliciting immune rejection *in vivo* (281, 282). Nevertheless, there is a rationale for combining ICB with strategies that promote TLS development. The presence of memory B cells and plasma cells may contribute to both T cell activation and antigen presentation, amplifying the local anti-tumor immunity through carefully coordinated B-T cell interactions.

### 5.3 Promising prospects in targeting EBV-driven cancers with immunotherapy

Satisfactory responses to ICBs have been reported in metastatic NPCs (243, 283, 284). From an experimental perspective, perhaps the way forward is to study immune responses to novel treatments such as EBV-targeted cell therapies and cancer vaccines. A case report highlighted the potential synergy of ICBs and the adoptive transfer of EBV-specific T cells resulting in the patient showing complete resolution of metastatic disease without any signs of relapse. More interestingly, the combinatory treatment resulted in the emergence of novel T cell clonotypes alongside the maintenance of dominant clones, indicating potential epitope spreading and TCR diversification (285). It was also demonstrated that the use of CRISPR/Cas9 to delete PD-1 could enhance *in vitro* and *in vivo* killing of GCs by cytotoxic T lymphocytes (CTLs) specific for the viral antigen LMP2A (latent membrane protein 2A) (251). Prior immunization with BARF1, an EBV antigen presented on tumor cells, may represent a potential tumor vaccine target, eliciting both humoral and T cell-mediated immune responses *in vivo* (286). Additionally, the targeting of alternative immune checkpoints such as TIM-3 and LAG-3 on tumor-specific CTL clones can further enhance eradication of tumor cells as demonstrated in GCs (287).

Alternatively, one could also consider exploiting the fact that EBV-infected targets are highly susceptible to NK cell-mediated killing (288, 289). NK cells incubated in EBV seropositive serum were demonstrated to be highly activated *in vitro*, suggesting an interplay of the humoral immunity or other upregulated cytokine factors within the viral-infected host (290). Adoptive transfer of NK cells in combination with anti-PD1 therapy also showed promising efficacy in a GC xenograft model (291). Furthermore, mesothelin-targeting CAR-NK92 cells were demonstrated to specifically eradicate GCs both *in vitro* and *in vivo* (292). Still, it is well known that adoptive NK cell therapies in general do not penetrate well into solid tumors (293, 294). It may perhaps be more effective to reinvigorate intratumoral NK cells within the TME, but our current understanding of tumor-infiltrating NK cells in GCs and NPCs are limited. The upregulation of EBV-encoded BZLF1 (BamHI Z fragment leftward open reading frame 1) during the early lytic cycle sensitizes viral-infected cells to NK cell-mediating killing by the upregulation of NKG2D ligands. However, such BZLF1-dependent sensitization could be counteracted by BHRF1, a viral homologue for BCL-2 acting as a potent anti-apoptotic protein also known to drive chemoresistance in EBV-associated cancers (295, 296). Apart from BHRF1-conferred resistance, other immune evasion pathways were characterized particularly in EBVaNPC. EBV-encoded microRNA BART7 (miR-BART7) indirectly represses the expression of NKG2D ligand, MIC-A (major histocompatibility complex class I chain-related peptide A) to desensitize NPC tumors from NK cell-mediated killing (297). The EBV gene, BCRF1, also encodes an IL-10 homologue that was demonstrated to impair NK cell and CD4 T cell activity (272). Another study reported lower infiltrates of granzyme B-positive NK cells in EBVaNPCs and further demonstrated that LMP2A upregulates F3 (Coagulation factor III), which in turn triggers platelet aggregation that suppresses NK cell cytotoxicity (298). Despite EBVaGCs being less extensively studied than NPCs, an immune deconvolution on the TCGA dataset for bulk GC tumor transcriptomics data putatively uncovered EBVaGCs to have higher infiltration of NK cells and T cells, but unexpectedly not B cells, as compared to EBV-negative GCs. Compared to both adjacent normal and EBV-negative tissues, EBVaGCs expressed higher levels of CD155 (encoding PVR) which is a ligand for either inhibitory receptors (TIGIT and CD96) or activating receptor, DNAM-1 (299, 300). Thus, future studies can focus on characterizing the immune profile of intratumoral NK cells to evaluate the potential of existing anti-TIGIT blockade as an alternative ICB (301, 302), acting to potently reinvigorate NK cell activity by enhancing PVR-DNAM1 binding.

Looking forward, one can also leverage on the wealth of publicly accessible NPC datasets to study novel cellular interactions that may play a critical role within the tumor immune landscape. Large datasets can be interrogated using immune deconvolution approaches such as CIBERSORT to uncover representative TME features that are prognostic to patient survival (303, 304). Single cell transcriptomics also revealed a unique population of Clec9a+ DCs, though its functions and relevance in NPCs remained unelucidated (278). In addition, the use of the CellPhoneDb algorithm further revealed putative cell-cell interactions that is unique between LMP+ NPC tumor cells and immune cells, driven by the chemokine CX3CL1 (305, 306). Contrastingly, there are lesser data resources available to understand EBVaGC. One can explore the use of a transplantable strain of EBVaGC known as “KT” in a humanized mice xenograft (307). Taken together, there are indeed several EBV-derived molecules that are of druggable potential but yet, a research gap remains to address if these targets can be feasibly combined with ICBs to yield better therapeutic responses in the clinics. We also render that EBVaNPCs may seem to appear more immune-tolerant for the fact that they are much more well-studied than EBVaGCs, which are of much lower incidence rates. Although not yet elucidated, it is highly plausible that EBV-driven mechanisms of immune evasion could occur in EBVaGCs as similarly observed in NPCs.

## 6 Concluding remarks

In general, it appears that cancers of viral etiologies tend not to rely on tumor mutational burden or neo/tumor antigens to prime immune responses. Evidently in these viral-driven cancers, ICB treatments are widely evaluated in the clinics while viral-associated immunity can be exploited in various forms of tumor vaccines or adoptive cell therapies (Summarized in **Table 1**). Taken together, we should agree here that there is substantial knowledge to exploit viral-associated immunogenicity and the highly dynamic TME for translation into direct applications within the oncology space. Moreover, the utilization of artificial intelligence (AI) has enabled precise prediction of ICB response (309), tumor progression or recurrence (132, 310), as well as immunogenic neoantigens (311, 312) for novel immunotherapies such as neoantigen vaccines. With a multi-omics, AI-powered analysis of the TME heterogeneity (313), it is hoped that immune signatures of different viral-associated cancers can be further delineated, ultimately improving therapeutic efficacy and patient outcomes with precision immuno-oncology. To conclude, we envision that clinicians can recognize and leverage these factors as powerful biomarkers for patients’ responses to ICBs, and at the same time, inspire future science to revisit rare cancers such as MCPyV+ MCCs and EBVaGCs for more novel and critical discoveries to improve conventional ICBs.

**Table 1:** Non-exhaustive examples highlighted for various immunotherapies targeting viral-associated cancers.

Cancer type	Virus	In clinics	Experimental
Merkel Cell Carcinoma (MCC)	MCPyV	• Phase 2 clinical trial with Pembrolizumab (44) • Pembrolizumab on CITN-09/Keynote-017 trial (33) • Meta-analysis for PD-1/PD-L1 treatments in MCC patients (45) • A case study reporting adoptive transfer of polyomavirus- specific T cells (35)	• *In vitro* expansion of MCPyV-specific T cells and demonstrated cytotoxicity (45) • Use of HDAC inhibitors to enhance antigen presentation (37) • STING agonism to enhance cytokine production and T cell immunity *in vitro* (38) • DNA vaccines targeting large T/small T antigen (49, 50, 53) • MCPyV-LT antigen-primed dendritic cells as potential DC vaccine (51)
Hepatocellular Carcinoma (HCC)	HBV, HCV	• Phase 3 clinical trials involving Atezolizumab (110, 115) • Phase 3 clinical trial for Tremelimumab + Durvalumab (111) • Phase 3 clinical trial for Lenvatinib + Pembrolizumab (116) • Clinical trials using oncolytic virus, Pexa-Vec (117–119) • Case reports for HbsAg-specific TCR T cell therapy (71, 72) • Phase 1 clinical trial for patients receiving short-lived HBV- specific T cell therapy (70)	• HBx silencing with siRNA enhances activity of CD8 T cells and NK cells (98) • Anti-TREM2 mAb targets macrophages and improves responsiveness to anti-PD-1 in mice (120, 121) • GPC3-targeting CAR T cell therapies in HCC (101–103) • GPC3-targeted CAR NK therapy (104, 105) • Co-blockade of TIGIT/PD-1 restores ex-vivo functions of CD8 TILs in HCC (68)
HPV-associated Cancers (Cervical, HNSCC)	HPV16, HPV18	• Clinical trials involving Pembrolizumab (143, 144) • Clinical trials involving Nivolumab (145, 146) • Clinical studies involving ADXS11-001 (Vaccine targeting HPV-E7 antigen) (237, 239–241) • Phase 1 clinical trial for TCR T cell therapy targeting HPV-E7 in cervical cancer and HNSCCs (234)	• Adoptive NK cell transfers *in vitro* and HNSCC xenografts (208) • *In vitro* efficacy of CD70-targeting CAR T cells in HNSCCs (308) • Dendritic cell-tumor cell fusion as a DC vaccine against murine SCC *in vivo* (233) • *In vivo* efficacy HPV-E7 targeted TCR-T cell therapy in cervical cancer (235) • Combination of CAR T cells and HPV-E7 primed DCs targeting cervical cancer (232)
EBV-Associated Cancers (NPC, EBVaGC)	EBV	• Cross-sectional analysis of NPC patients involving immune checkpoint inhibitors and cell therapies (243) • Phase 2 multicenter consortium of NPC patients receiving Nivolumab (283) • Phase 2 clinical trial for EBV-NPC patients receiving a combination of Nivolumab and Ipilimumab (284) • Case report of an NPC patient receiving adoptive transfer of EBV-specific T cells and Nivolumab (285) • Case report of benefit from avelumab despite low TMB in EBVaGC (254)	• DNA vaccine targeting BARF1 *in vivo* (286) • CRISPR-mediated deletion of PD-1 in LMP2A-specific T cells targeting EBVaGC (251) • Combination of anti-PD-1 and adoptive NK cell transfer targeting GC *in vivo* (291) • Efficacy of mesothelin-specific CAR-NK92 cells demonstrated in xenograft models of GCs (292) • Combinatory targeting TIM-3/LAG-3/TIGIT as alternative immune checkpoint *in vitro* (287) • Inhibition of F3-mediated platelet aggregation reinvigorates NK cell activity in NPC and EBVaGCs (298)
